# Thermodynamic stabilities of three-way junction nanomotifs in prohead RNA

**DOI:** 10.1261/rna.059220.116

**Published:** 2017-04

**Authors:** Alyssa C. Hill, Susan J. Schroeder

**Affiliations:** 1 Department of Microbiology and Plant Biology; 2 Department of Chemistry and Biochemistry, University of Oklahoma, Norman, Oklahoma 73019, USA

**Keywords:** prohead RNA, packaging RNA, three-way junction, thermodynamics, RNA self-assembly, RNA nanotechnology

## Abstract

The thermodynamic stabilities of four natural prohead or packaging RNA (pRNA) three-way junction (3WJ) nanomotifs and seven phi29 pRNA 3WJ deletion mutant nanomotifs were investigated using UV optical melting on a three-component RNA system. Our data reveal that some pRNA 3WJs are more stable than the phi29 pRNA 3WJ. The stability of the 3WJ contributes to the unique self-assembly properties of pRNA. Thus, ultrastable pRNA 3WJ motifs suggest new scaffolds for pRNA-based nanotechnology. We present data demonstrating that pRNA 3WJs differentially respond to the presence of metal ions. A comparison of our data with free energies predicted by currently available RNA secondary structure prediction programs shows that these programs do not accurately predict multibranch loop stabilities. These results will expand the existing parameters used for RNA secondary structure prediction from sequence in order to better inform RNA structure–function hypotheses and guide the rational design of functional RNA supramolecular assemblies.

## INTRODUCTION

Multibranch loops are key determinants of structural and functional roles in RNA. In prohead RNA (pRNA), an essential component of the phi29-like bacteriophage DNA packaging motor (Guo et al. 1987), the three-way junction (3WJ) is a multibranch loop that is a flexible, dynamic region in the RNA (Lescoute and Westhof 2006; Zhang et al. 2012, 2013) that helps to correctly place helices in the spatial orientation necessary for packaging (Zhang et al. 1997; Hoeprich and Guo 2002; Ding et al. 2011; Hill et al. 2016). In vitro, pRNA self-assembles, with some sequences capable of forming dimers, trimers, and higher-order multimers depending on the assembly conditions used. Recent studies have shown that the SF5 and M2 pRNAs have the highest propensities for in vitro self-assembly (Gu and Schroeder 2011; Hao and Kieft 2014). The shape, self-assembly properties, and stability of pRNA can be leveraged in the rational design of functional supramolecular structures, including polyvalent nanoscale delivery systems (Chen et al. 2000; Shu et al. 2004, 2011b, 2013; Guo 2005; Guo et al. 2005, 2006; Li et al. 2009, 2015a; Tarapore et al. 2011; Haque et al. 2012; Khisamutdinov et al. 2014). However, an important consideration in the design of functional nanostructures is the stability of the pRNA “building block” (Shu et al. 2011a; Li et al. 2015a,b).

Different pRNAs share only 12% sequence similarity, but pRNA secondary structure and function are conserved (Gu and Schroeder 2011). Although different pRNAs have different interlocking loop sequences and therefore different interlocking loop stabilities, the overall energetics for pRNA dimerization are also conserved, suggesting that another pRNA nanomotif plays a compensatory role in stabilizing the RNA (Gu and Schroeder 2011). Studies on designer pRNA sequences, which retained only the wild-type (WT) interlocking loops and 3WJ sequences but changed the sequences in the Watson–Crick pairs, indicated that helices do not contribute to pRNA assembly in a sequence-dependent fashion (Ding et al. 2011). Studies that mixed pRNA interlocking loops and 3WJs revealed similar insights, demonstrating that mediocre loop–loop interactions can be overcome with highly stable scaffolds (Hao and Kieft 2014). These results implicate the 3WJ as an important contributor to pRNA self-assembly.

**FIGURE 1.**
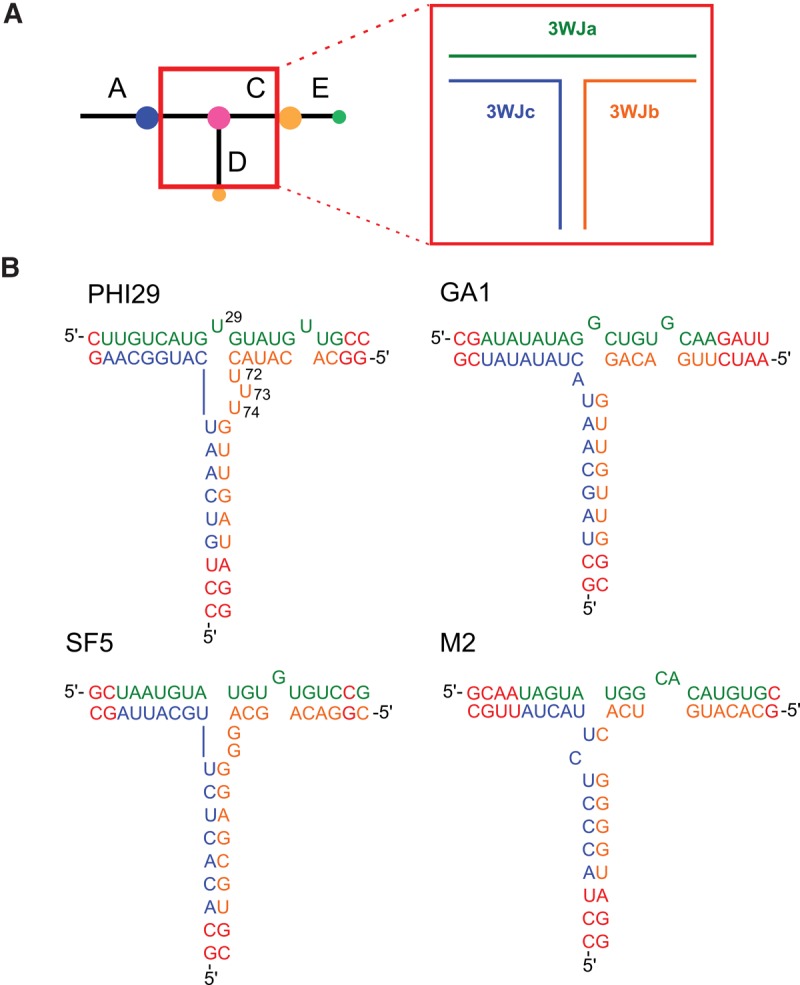
(*A*) Ball-and-stick model of pRNA, where balls represent loops and sticks represent helices. The pRNA 3WJ nanomotif, *inset*, comprises strands 3WJa (green), 3WJb (orange), and 3WJc (blue). (*B*) pRNA 3WJ constructs’ primary and secondary structures (Bailey et al. 1990) where strand 3WJa is in green, 3WJb is in orange, and 3WJc is in blue. Changes to WT sequences are indicated in red. Nucleotides deleted in the phi29 pRNA 3WJ are labeled.

Using a three-component RNA system designed for UV optical melting (Fig. 1; Diamond et al. 2001; Liu et al. 2011), we measured the thermodynamic parameters for four natural pRNA 3WJs and seven mutated phi29 pRNA 3WJs. Here, we report the thermodynamic stabilities for these pRNA 3WJ sequences. Our results show that the GA1, SF5, and M2 pRNA 3WJs are more stable than the WT phi29 pRNA 3WJ commonly used as a scaffold in RNA-based nanotechnology. Furthermore, we show that certain deletions at the phi29 pRNA 3WJ increase its stability relative to WT. Finally, we show that metal ions have a differential stabilizing effect on pRNA 3WJs, and we demonstrate the need for improved RNA secondary structure stability predictions for multibranch loops. This work extends our knowledge of the biophysical properties of pRNA and provides a foundation for further investigation into the viability of other, underexplored pRNA 3WJ nanomotifs in the rational design of functional RNA supramolecular structures.

## RESULTS

### pRNA 3WJ nanomotif stabilities

Thermodynamic parameters determined by UV optical melting for each construct and its respective 3WJ nanomotif are reported in Table 1. The GA1, SF5, and M2 pRNA 3WJs showed greater stability than the phi29 pRNA 3WJ (Table 1).

**TABLE 1.**
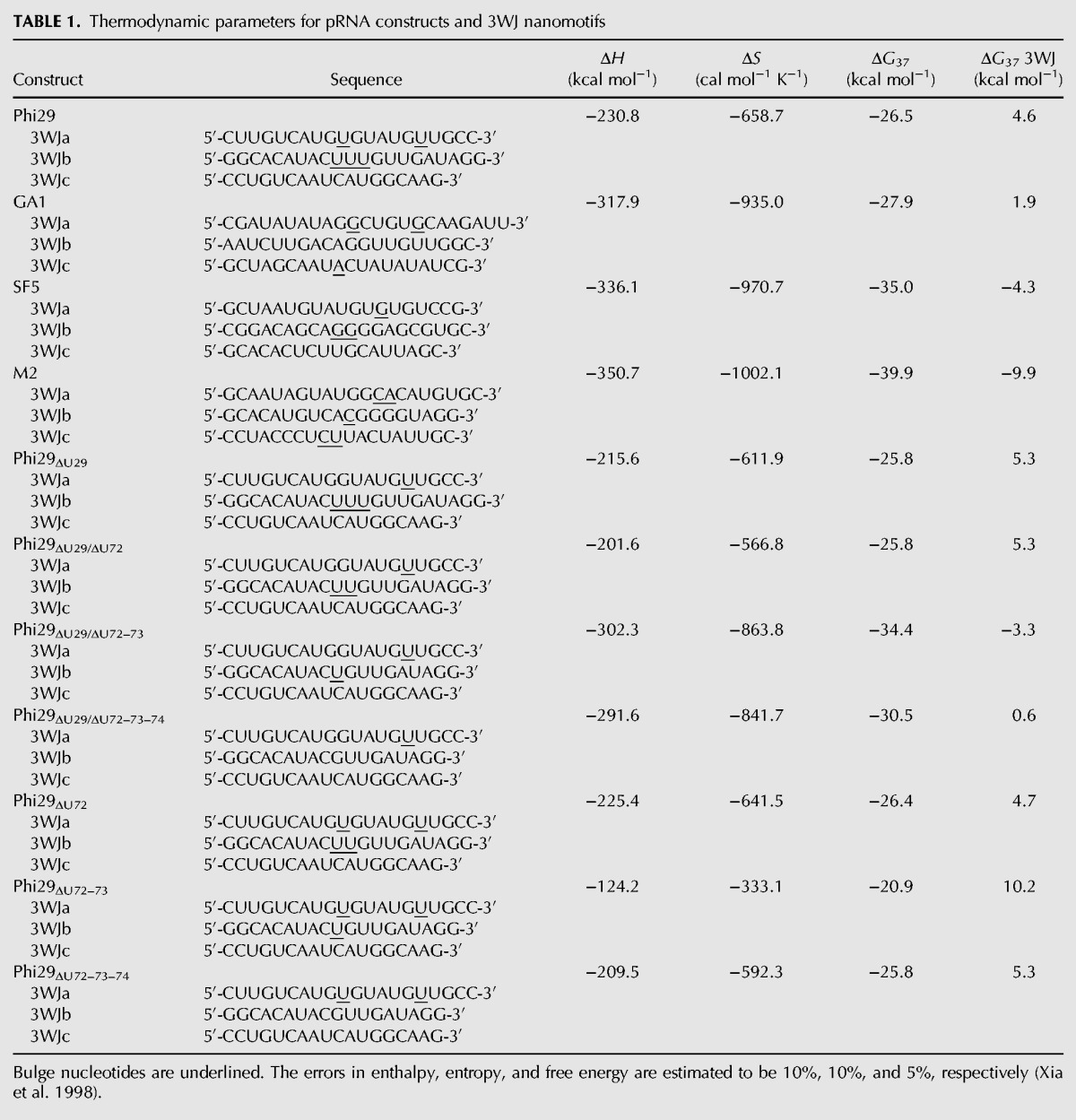
Thermodynamic parameters for pRNA constructs and 3WJ nanomotifs

**FIGURE 2.**
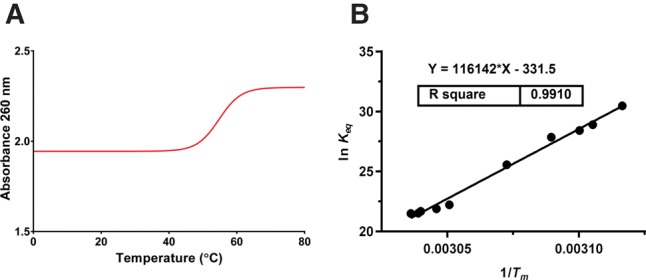
(*A*) Nonlinear melt curve fit (*R*^2^ > 0.99) for the WT phi29 3WJ construct collected at 31.2 µM showing a sharp, cooperative transition. Data points were collected at a rate of 6/s with a heating rate of 1°C/min. (*B*) van't Hoff plot of phi29 3WJ melt data (*R*^2^ > 0.99), where the slope is −Δ*H*/*R* and the *y*-intercept is Δ*S*/*R*. Data were fit using the Marquardt–Levenberg method in Meltwin (McDowell and Turner 1996).

Melt curves showed very sharp transitions, supporting the assumption of a cooperative transition from the RNA triplex to single-stranded RNAs (Fig. 2A). None of the single strand or pairwise combination melts showed a significant transition that would compete with the 3WJ (Supplemental Figs. S1, S2). For example, a melt of phi29 strands 3WJa and 3WJb showed a transition with a melting temperature of 51°C, while the complete 3WJ (i.e., strands 3WJa, 3WJb, and 3WJc together) showed a transition with a melting temperature of 56°C (Supplemental Fig. S2). However, formation of the 3WJ is favored when all three strands are present. As previously shown for non-self-complementary RNA duplexes, the non-self-complementary duplex will form even when the *T*_m_ for an alternative conformation of a single strand forming a self-complementary duplex has a higher *T*_m_ if the enthalpy is more favorable for the non-self-complementary duplex (Supplemental Table S3; Schroeder and Turner 2000). Free energies for the constructs and their respective 3WJs were calculated from van't Hoff plots, where the goodness of linear fit was ≥0.90 for all melts (Fig. 2B). The free energies for the investigated 3WJs range from −9.9 kcal/mol to 10.2 kcal/mol. By comparison, 1.4 kcal/mol at 37°C is approximately one order of magnitude in a binding constant. Thus, the range of free energies of formation for the investigated 3WJs spans 14 orders of magnitude in terms of binding constants.

The phi29 pRNA 3WJ is stabilized when certain uridine (U) residues are deleted from the junction. Specifically, the following two deletion combinations increased stability relative to the WT phi29 pRNA 3WJ: (i) deletion of a single U bulge (U29) in strand 3WJa along with two of the three U residues in the tri-U bulge in strand 3WJb (i.e., U72–73–74); and (ii) deletion of all bulge U residues at the 3WJ (i.e., U29/U72–73–74) (Table 1; Fig. 1B; Zhao et al. 2012). Other investigated deletions either did not significantly affect the stability of the phi29 pRNA 3WJ or actively destabilized it (Table 1).

**FIGURE 3.**
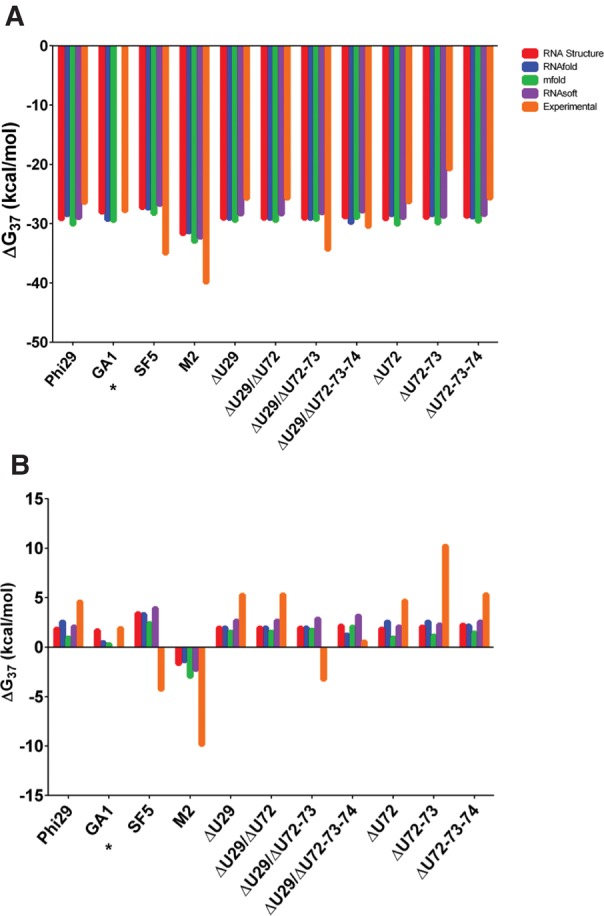
(*A*) Measured thermodynamic stabilities and secondary structure free-energy predictions for pRNA 3WJ constructs using RNA Structure (Mathews et al. 2004), RNAfold (Lorenz et al. 2011), mfold (Zuker 2003), and RNAsoft (Andronescu et al. 2003). (*B*) Calculated 3WJ nanomotif thermodynamic stabilities and secondary structure free-energy predictions using the same programs. *RNAsoft did not output a secondary structure free energy for the GA1 pRNA 3WJ due to computer run-time limitations.

Of the four RNA secondary structure prediction programs used, none accurately predicted either the actual free energies of the junctions or variations in phi29 pRNA mutant 3WJ stabilities (Fig. 3; Supplemental Table S1). Best predictions ranged from within 1 kcal/mol of the measured free energy (for phi29ΔU29/ΔU72–73–74, by RNAsoft) to 9 kcal/mol (for phi29ΔU72–73, by RNAfold), whereas worst predictions ranged from within 1 kcal/mol (for phi29ΔU29/ΔU72–73–74, by RNAsoft) to 11 kcal/mol (for phi29ΔU72–73, by RNAfold). At best, the prediction programs were off by an average of 4 (±2) kcal/mol from measured free energies.

### Metal-ion binding

**FIGURE 4.**
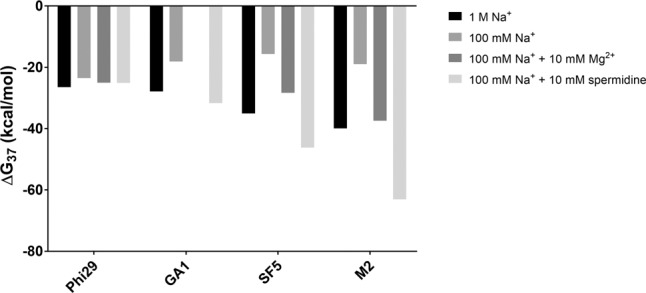
Metal-ion effects on natural pRNA 3WJ constructs. Optical melts of the GA1 3WJ in 100 mM Na^+^ and 10 mM Mg^2+^ did not meet the van't Hoff plot goodness of linear fit cutoff criterion of ≥0.90.

The effects of Na^+^, Mg^2+^, and spermidine (a 3^+^-charged species) on pRNA 3WJ stabilities are depicted in Figure 4. Complete thermodynamic parameters are provided in Supplemental Table S2. Relative to 100 mM NaCl, the phi29 pRNA 3WJ construct was nearly equally stabilized by Mg^2+^ and spermidine, while the GA1, SF5, and M2 pRNA 3WJs were differentially affected by these ions. Addition of Mg^2+^ stabilized both the SF5 and M2 pRNA 3WJs. The addition of spermidine had an increasing stabilizing effect on the GA1, SF5, and M2 pRNA 3WJs, respectively (Fig. 4).

### Electrophoretic gel mobility shift assays

**FIGURE 5.**
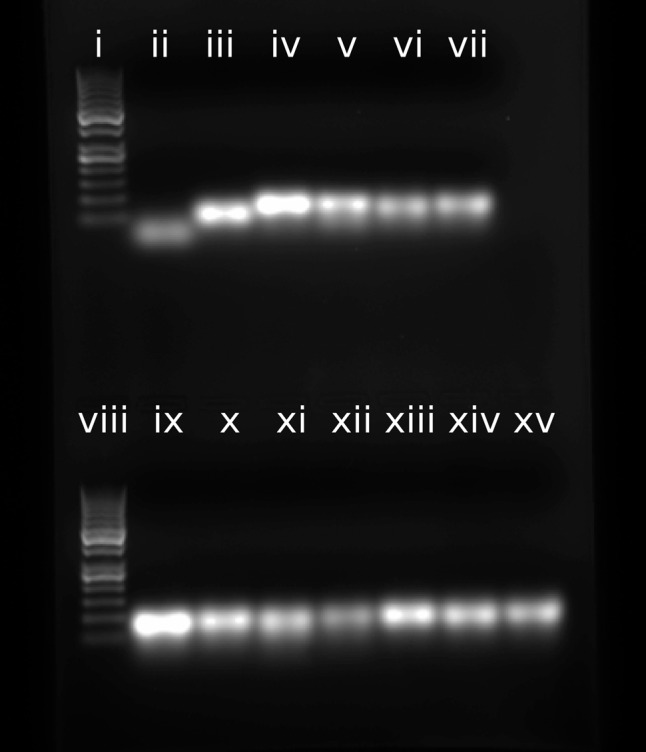
Gel mobility of 50-bp ladder (lanes *i* and *viii*), Phi29 strand 3WJa (lane *ii*), Phi29 strands 3WJa + 3WJb (lane *iii*), Phi29 3WJ (lane *iv*), GA1 3WJ (lane *v*), SF5 3WJ (lane *vi*), M2 3WJ (lane *vii*), Phi29ΔU29 (lane *ix*), Phi29ΔU29/ΔU72 (lane *x*), Phi29ΔU29/ΔU72–73 (lane *xi*), Phi29ΔU29/ΔU72–73–74 (lane *xii*), Phi29ΔU72 (lane *xiii*), Phi29ΔU72–73 (lane *xiv*), and Phi29ΔU72–73–74 (lane *xv*). Assembly was performed in standard melt buffer (1 M NaCl, 10 mM sodium cacodylate, 0.5 mM EDTA, pH 7) to confirm formation of the 3WJs under optical melting conditions. Phi29 strand 3WJa (lane *ii*) and strands 3WJa + 3WJb (lane *iii*) were included as references. All 3WJs run at approximately the same rate.

A gel depicting the mobility of all pRNA 3WJs under standard melt buffer conditions (1 M sodium chloride, 10 mM sodium cacodylate, 0.5 mM EDTA, pH 7.0) relative to a single strand (phi29 3WJa) and a pairwise combination (phi29 3WJa + 3WJb) is shown in Figure 5. All assembled 3WJs run at approximately the same rate. Additionally, gels depicting the mobility of each pRNA 3WJ construct relative to each single RNA strand and all pairwise combinations in TMS buffer (50 mM Tris–HCl, pH 7.8, 100 mM NaCl, 10 mM MgCl_2_) are shown in Supplemental Figure S3 for the purpose of comparison to previous work published on the assembly and stabilities of various biological RNAs (Shu et al. 2011a). For each gel, mobility decreased as more components of the RNA system were added. Single strands showed the fastest migration and pairwise combinations showed intermediate migration relative to a slow-migrating band that appeared when all three RNA 3WJ strands were present (Fig. 5; Supplemental Fig. S3), indicating that the three RNA components interact more favorably than any two components, and confirming formation of the pRNA 3WJ from strands 3WJa, 3WJb, and 3WJc.

## DISCUSSION

### 3WJ stabilities in relation to loop–loop interaction stabilities and self-assembly

**FIGURE 6.**
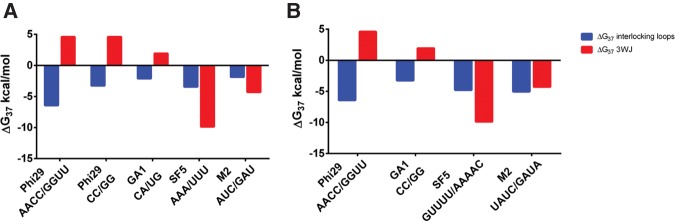
Experimental 3WJ data presented with interlocking loop stabilities calculated using the Nearest Neighbor Database (Turner and Mathews 2010) of sequences reported by (*A*) Gu and Schroeder (2011) and (*B*) Hao and Kieft (2014). Interlocking loop sequences are provided for each pRNA. Both the experimental stability data and the calculated stability data were determined for RNA in 1 M NaCl.

Among the investigated pRNA 3WJ constructs, the SF5 and M2 pRNA 3WJs were most thermodynamically stable, making them attractive alternatives to the phi29 pRNA 3WJ scaffold used in pRNA-based nanotechnology. Interestingly, these pRNAs also have shown the highest propensity for self-assembly under the laboratory conditions studied thus far (Gu and Schroeder 2011; Hao and Kieft 2014). Analyses of our measured 3WJ stabilities with loop–loop interaction stabilities calculated from sequences provided by Gu and Schroeder (2011) and Hao and Kieft (2014) provide insights into whether the 3WJ nanomotif plays a compensatory role in stabilizing pRNA. The analyses indicated that while loop–loop interactions are all favorable, 3WJs have a wider range of stabilities (Fig. 6). Specifically, the phi29 and GA1 pRNAs have unfavorable 3WJ stabilities, while the M2 and SF5 pRNAs have favorable 3WJ stabilities (Fig. 6). For phi29 and GA1 pRNAs, the loop–loop interaction stabilities are offset by 3WJ stabilities, while for SF5 and M2 pRNAs, the loop–loop interactions and the 3WJ are both stabilizing. The combination of stabilizing nanomotifs may help explain why only SF5 and M2 pRNAs have shown in vitro self-assembly of higher-order multimers (Gu and Schroeder 2011; Hao and Kieft 2014). Recently, the M2 pRNA 3WJ was shown to favor a conformation that promotes higher-order multimer assembly (Hao and Kieft 2016). Furthermore, a chimeric pRNA where the phi29 3WJ was placed in the M2 architecture showed reduced assembly (Hao and Kieft 2016). Consistent with their increased thermodynamic stabilities and propensities to assemble into higher-order multimers relative to phi29, the SF5 and M2 sequences may both adopt 3WJ preorganizations that do not require the disruption of existing coaxial stacking or other favorable interactions in order to self-assemble.

In a recent study (Li et al. 2015b), a phi29 pRNA-derived construct retaining all U residues at the 3WJ was shown to be thermodynamically stable. Our results can be used to improve the efficiency of self-assembly in this system with specific U deletions at the 3WJ that increase its stability. Here we show that the ΔU29/ΔU72–73 and ΔU29/ΔU72–73–74 deletions at the phi29 pRNA 3WJ are stabilizing (Table 1), suggesting alternatives to WT phi29 pRNA scaffolds in the rational design of functional RNAs. Although no clear pattern emerges for any correlation between the number of nucleotides at the 3WJ and the stability of the 3WJ, the deletions may affect helix stacking such that favorable stacking stabilizes the junction, whereas unfavorable stacking destabilizes the junction. Favorable stacking may account for some, but not all, of the stability differences observed. For example, free energies of helix stacking in a two-component 3WJ construct range from −3.4 to −5.2 kcal/mol (Liu et al. 2011). A previous packaging study (Zhao et al. 2012) on phi29 pRNA 3WJ deletion mutants reported that deletion of any two of the four bulge U residues at the junction (i.e., U29/U72–73–74) supports pRNA binding to the viral prohead as well as WT levels of DNA packaging. In vivo, it is possible that individual pRNAs are further stabilized by their interactions with other pRNAs, proteins, or metal ions.

### Structure–energetics relationships in the pRNA 3WJ

Two crystal structures have captured snapshots of the structures of the phi29 pRNA 3WJ. The two crystal structures have different space groups, crystal packing, resolution, helix orientations, sequence modifications, and metal-ion binding sites but share in common the coaxial stacking of helices A and D (Fig. 1A). A truncated phi29 construct with interlocking loops at 3.5 Å shows metal ions bound near the GU closing pair of the 3WJ in the native sequence (Ding et al. 2011). A three-component model for the phi29 pRNA 3WJ motif at 3.05 Å was stabilized by two divalent metal ions binding between two helices (Zhang et al. 2013). Comparison of the two metal-ion bound structures (Ding et al. 2011; Zhang et al. 2013) indicates a change in the A and D interhelical angle and helix distortion where the metal ions bind (Zhang et al. 2013). EPR methods determined another multibranch loop conformation in solution in a pRNA dimer in the presence of Mg^2+^ (Zhang et al. 2012). Single-molecule FRET analysis revealed the dynamic structural changes in this 3WJ when Mg^2+^ was added (Zhang et al. 2013). Despite the differences in techniques and constructs, metal-ion binding at the 3WJ is a common feature in all of these structural studies.

Our thermodynamic data on constructs based on the three-component model of the 3WJ show that Mg^2+^ and spermidine (an aliphatic amine and trivalent ion) nearly equally stabilize the phi29 3WJ (Fig. 4; Supplemental Table S2). An important consideration when comparing our data to the construct crystallized with divalent metal ions is a mutation in one of the four nucleotides shown to chelate the ions. In the crystallized construct, nucleotides G23/A90 and C24/A89 were shown to bind one divalent ion each (Zhang et al. 2013). However, the cytidine at position 24 (C24) was mutated from the uridine found in WT phi29 pRNA and in the constructs used in this study (Fig. 1B; Zhang et al. 2013). Metal ions may help stabilize different sequences in different ways, but they may ultimately serve a similar structural and energetic role.

**FIGURE 7.**
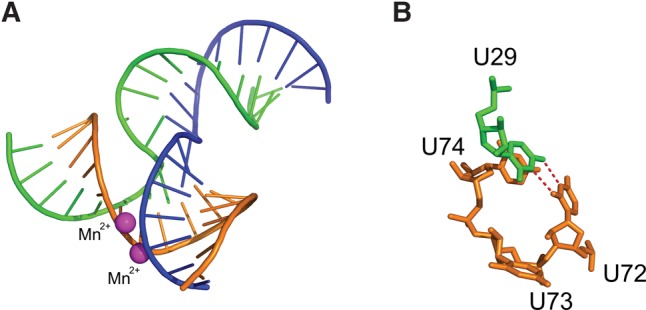
phi29 pRNA 3WJ structure (Zhang et al. 2013). (*A*) 3WJa is in green, 3WJb is in orange, and 3WJc is in blue. Bound divalent metal ions (Mn^2+^) are in magenta. (*B*) Bulge residues at the 3WJ (i.e., U29/U72–73–74) are shown as sticks. A *cis* base pair forms between the Watson–Crick edges (Leontis et al. 2002) of U29 and U72. U29 and U74 form a base stack.

The crystallized 3WJ core and the new thermodynamic data presented here provide a foundation for inferences about RNA structure–energetics relationships, especially for the four bulge U residues at the 3WJ. Non-Watson–Crick base-pairing predominates in RNA 3D structure, forming motifs that facilitate RNA–RNA interactions and bind ligands (Leontis et al. 2002; Westhof et al. 2011). The phi29 3WJ crystal structure (Zhang et al. 2013) revealed the formation of a *cis* base pair between the Watson–Crick edges of U29 and U72 as well as base stacking between U29 and U74 (Fig. 7B; Leontis et al. 2002; Zhang et al. 2013). By comparison, the phi29 pRNA 3WJ constructs in which U29 and at least one of the three U residues in the tri-U bulge (i.e., U72–73–74) were retained did not appear to have equivalent thermodynamic stabilities (Supplemental Table S1), despite the possibility of base pair formation across these constructs’ 3WJs. However, the stability of a 3WJ where U29 and two of the three U residues in the tri-U bulge were retained was nearly equal to that of the WT phi29 pRNA 3WJ (4.7 versus 4.6 kcal/mol, respectively). This could suggest that base pair formation and base stacking are preserved in this 3WJ construct. Interestingly, notable increases in the stability of the phi29 pRNA 3WJ occurred only when one of the U residues in the tri-U bulge was retained (−3.3 kcal/mol), or when all bulge U residues at the 3WJ were deleted (0.6 kcal/mol). Neither of these 3WJs would permit the observed base-pairing or base stacking between U residues across the junction, suggesting that pairing and stacking are not the only favorable tertiary interactions stabilizing the junction. These mutations may allow for different, favorable helical coaxial stacking interactions, which may contribute to the stability differences observed (Walter et al. 1994). Coaxial stacking in multibranch loops is very difficult to predict accurately, and it becomes more difficult with larger junctions that contain unpaired nucleotides (Tyagi and Mathews 2007). Thus, structural data on these constructs will be necessary to form a more complete picture of why some 3WJ sequences are more stable than others.

### Future improvements in predicting RNA 3WJ stabilities

Importantly, the free-energy outputs by RNA secondary structure prediction programs can be improved for multibranch loops. While all of the programs implemented in this study utilize the same free-energy database, the way that multibranch loops are predicted varies in different RNA structure prediction programs. The prediction may consider all possible conformations of coaxial stacking, include only the single most favorable coaxial stacking arrangement, or include knowledge-based parameters from analysis of known secondary structures (Mathews et al. 2004; Andronescu et al. 2010; Gruber et al. 2015; Kennedy 2016). None of the algorithms was able to account for the magnitude of differences observed among the measured stabilities of the investigated 3WJs. All programs overestimated the stabilities of the phi29 and GA1 pRNA 3WJ constructs and underestimated the stabilities of the SF5 and M2 pRNA 3WJ constructs (Fig. 3; Supplemental Table S1). Furthermore, none of the programs’ predictions discriminated between the stabilities of constructs with deletions at the phi29 pRNA 3WJ. For example, neither the mutant that was shown to have the highest stability, nor the mutant that was shown to have the lowest stability, was predicted as such (Fig. 3; Supplemental Table S1). Instead, deletion mutants were all predicted to have roughly the same free energies (within ∼1 kcal/mol). Compared to measured free energies, these predictions illustrate the need for refinement of the parameters used to make RNA secondary structure and free-energy predictions from sequence. Our work sets the foundation for further studies on multibranch loops of different sizes, asymmetries, and sequences in order to develop more robust prediction rules.

Prohead RNA (pRNA) is an important component of the phi29-like bacteriophage DNA packaging motor. Due to its stability and self-assembling properties in vitro, pRNA has been used successfully as a scaffold in the rational design of functional RNA supramolecular structures (Chen et al. 2000; Shu et al. 2004, 2011b, 2013; Guo 2005; Guo et al. 2005, 2006; Li et al. 2009, 2015a; Tarapore et al. 2011; Haque et al. 2012; Khisamutdinov et al. 2014). Compared to phi29 pRNA, the stabilities of other natural and mutated pRNA sequences remain relatively underexplored (Shu et al. 2011a; Li et al. 2015b). In this study, we investigated the thermodynamic stabilities of four natural and seven mutated pRNA 3WJ nanomotifs. We show that the GA1, SF5, and M2 pRNA 3WJs, as well as two phi29 pRNA mutant 3WJs, are more stable than the WT phi29 pRNA 3WJ.

The results of our approach to determining 3WJ stabilities will be used to improve RNA secondary structure prediction from sequence. 3WJs are ubiquitous in natural RNA secondary structures but remain poorly predicted. Our work sets a foundation for better understanding the rules that govern 3WJ thermodynamic stabilities. Additionally, knowledge that the SF5 and M2 pRNA 3WJs and two phi29 pRNA mutant 3WJs are more stable relative to the WT phi29 pRNA 3WJ provides a solid foundation for increasing the sequence diversity and fine-tuning the stabilities of pRNA building blocks in RNA-based nanotechnology.

## MATERIALS AND METHODS

### pRNA 3WJ construct design

The pRNA 3WJ can be assembled from three RNA oligomers mixed in equimolar concentrations (Shu et al. 2011a; Zhang et al. 2013; Binzel et al. 2014; Li et al. 2015b). In contrast to UV optical melting studies performed on RNA 3WJs using only two strands (Diamond et al. 2001; Liu et al. 2011), the constructs investigated in this study were assembled from three RNA strands designated 3WJa, 3WJb, and 3WJc (Fig. 1). Constructs were designed to encompass the 3WJ region of folded pRNA, excepting the A, CE, and D bulges, to form three helices of approximately equal free energies. The designed pRNA 3WJ constructs retained as much sequence identity to WT pRNA as possible, and changes to WT sequences were made distal to the 3WJ “core” where necessary (Fig. 1B) to ensure that the free energy of helix formation for each branch was within 0.1 kcal/mol. The predicted free energy for each branch duplex includes a helix initiation term that assumes two strands come together independently. This is an overestimation of the penalty of helix formation in the third branch because this branch should not have the same entropic penalty in the 3WJ. Thus, our calculated 3WJ free energies underestimate the stabilities of the 3WJs. Sequences for each construct are provided in Table 1. Oligonucleotides were purchased from IDT and prepared according to the manufacturer's instructions. Purity was confirmed to be >95% by ^32^P labeling and gel electrophoresis.

### UV optical melting

For each construct, the three RNA oligomers 3WJa, 3WJb, and 3WJc were mixed in equimolar concentrations spanning a 100-fold dilution range from 0.4 to 40 μM. UV optical melting was performed as described previously (Schroeder and Turner 2009), with variation in the analysis for a three-component RNA system (Marky and Breslauer 1987). Melts were carried out under standard melt buffer conditions (1 M sodium chloride, 10 mM sodium cacodylate, 0.5 mM EDTA, pH 7.0) (Gu et al. 2013) using a Beckman Coulter DU-800 spectrophotometer. Absorbances at 260 and 280 nm were measured as a function of temperature from 4°C to 90°C, both the largest measurable temperature range and the range for which the midpoint and the constructs’ expected *T*_m_s were approximately equal (Tellinghuisen 2006; Zhukov and Karlsson 2007). In order to form reproducibly the lowest energy structure, RNA samples were heated to 90°C and slowly cooled prior to melting or the addition of magnesium (Diamond et al. 2001). The sodium chloride concentration was reduced by an order of magnitude (Bloomfield et al. 2000) for melts with 10 mM metal ions, but all other conditions remained the same. Due to the presence of EDTA, the effective Mg^2+^ concentration may have been slightly <10 mM. For each construct, optical melts of single RNA strands 3WJa, 3WJb, and 3WJc and all pairwise combinations were also performed (Supplemental Figs. S1, S2). Melt curves were fit using Meltwin (McDowell and Turner 1996) in order to determine melting temperatures, and thermodynamic parameters were determined from van't Hoff plots where the equilibrium constant *K*_eq_ was given by

$$\frac{1}{{\left(C_{T}/2n\right)}^{n-2}},$$

where *C*_T_ = total strand concentration and *n* = 3 for a trimolecular dissociation reaction with equilibria involving non-self-complementary sequences (Marky and Breslauer 1987), and where goodness of linear fit, a good estimate of error, was ≥0.90 (Zhukov and Karlsson 2007). Derivation of the equilibrium constant is provided in Supplemental Information. The sharpness of the melting transition and the linearity of the van't Hoff plots suggest two-state melting; however, the assumption of Δ*C*_p_ = 0 is not rigorously followed. Although the enthalpies show some temperature dependence, there is a large range of error in the enthalpy and heat capacity values. The errors in enthalpy and entropy are correlated, and thus, the free energy still provides a useful, predictive value. Furthermore, corrections for temperature-dependent changes in heat capacity have a small effect within error of the value for the final free energy of the multibranch loop motif, i.e., <0.5 kcal/mol (Diamond et al. 2001; Mathews and Turner 2002). Thermodynamic stabilities of the pRNA 3WJ nanomotifs were calculated by subtracting the stability contributions of the RNA helices as calculated from the Nearest Neighbor Database (Table 1; Fig. 3B; Turner and Mathews 2010).

### pRNA 3WJ secondary structure and free-energy predictions

The secondary structure and stability of each pRNA construct was predicted using four RNA secondary structure prediction programs: RNA Structure (Mathews et al. 2004), RNAfold (Lorenz et al. 2011), mfold (Zuker 2003), and RNAsoft (Andronescu et al. 2003). For predictions in RNA Structure, RNAfold, and mfold, pRNA strands 3WJa and 3WJb as well as 3WJb and 3WJc were joined with a 5′-aaaa-3′ hairpin and then the construct was folded as a single strand. Free-energy predictions were not affected by the position of the hairpins, i.e., whether the hairpins were placed between strands 3WJa and 3WJb and strands 3WJb and 3WJc, between strands 3WJb and 3WJc and strands 3WJc and 3WJa, or between strands 3WJc and 3WJa and strands 3WJa and 3WJb. For predictions in RNAsoft, pRNA strands 3WJa and 3WJb were joined with a 5′-aaaa-3′ hairpin and folded with strand 3WJc. Again, free-energy predictions were not affected by the position of the hairpin, i.e., whether the hairpin was placed between strands 3WJa and 3WJb, between strands 3WJb and 3WJc, or between strands 3WJc and 3WJa. For each construct, the most stable structure output by each program was the designed structure. No forced base pairs or single strand constraints were used. To correct for the added hairpins, two 5′-aaaa-3′ hairpin penalties were subtracted from the secondary structure stabilities output by RNA Structure, RNAfold, and mfold (Supplemental Table S1). For stabilities predicted by RNAsoft, one 5′-aaaa-3′ hairpin penalty was subtracted (Supplemental Table S1; Turner and Mathews 2010). Because the calculations made in Supplemental Table S1 take into account the initiation terms for the 3WJs, their free energies are accurate within error whether in the context of a single-stranded RNA, a duplex, or a three-component system. The entropic penalties for bringing together two oligomers are included in the free energy of initiation terms. The free energies of initiating an RNA hairpin and RNA intermolecular interactions are 5.4 ± 0.2 kcal/mol to 6.4 ± 0.2 kcal/mol (spanning loop lengths *n* = 3 to *n* = 9) and 4.09 ± 0.2 kcal/mol, respectively (Turner and Mathews 2010). Thus, while there is a substantial entropic energy difference in bringing together three oligonucleotide strands compared to a single strand self-folding, the calculation of the free energy of the multibranch loop accounts for this difference and makes comparison of the 3WJ motif comparable in different contexts.

### Electrophoretic gel mobility shift assays

Formation of the pRNA 3WJ constructs was monitored using electrophoretic gel mobility shift assays (EMSAs). RNA at a concentration of 40 μM in standard melt buffer (1 M sodium chloride, 10 mM sodium cacodylate, 0.5 mM EDTA, pH 7.0) was heated to 80°C for 10 sec and then cooled to 4°C at a rate of 0.1°C/sec in an MJ Research PTC-200 Peltier Thermal Cycler. The RNA was mixed with sucrose loading dye and run in TAE buffer at 50 V and 4°C in precooled, 2% (w/v) agarose stained with ethidium bromide. Mobility of a single RNA strand (phi29 3WJa), a pairwise combination (phi29 3WJa + 3WJb), and all assembled pRNA 3WJs were monitored. RNA at a concentration of 10 μM in TMS buffer (50 mM Tris–HCl, pH 7.8, 100 mM NaCl, 10 mM MgCl_2_) was heated to 80°C for 10 sec and then cooled to 4°C at a rate of 0.1°C/sec in an MJ Research PTC-200 Peltier Thermal Cycler. The RNA was mixed with sucrose loading dye and run in TAE buffer at 100V and 4°C in precooled, 4% (w/v) agarose stained with ethidium bromide. Mobility of each single RNA strand, pairwise combinations, and the pRNA 3WJ construct were monitored.

## SUPPLEMENTAL MATERIAL

Supplemental material is available for this article.

## COMPETING INTEREST STATEMENT

A.C. Hill and S.J. Schroeder are inventors listed on a provisional patent application filed through the Board of Regents for the University of Oklahoma in March 2016.

## Supplementary Material

Supplemental Material
