# Supplementary material for: Thermodynamic stabilities of three-way junction nanomotifs in prohead RNA
Source: RNA. 2017 Apr;23(4):521–9. doi: 10.1261/rna.059220.116 (PMC5340915; doi:10.1261/rna.059220.116)
Supplement: Supplemental Material [file supp_23_4_521__index.html]

Thermodynamic stabilities of three-way junction nanomotifs in prohead RNA — Supplemental Material 

# Thermodynamic stabilities of three-way junction nanomotifs in prohead RNA

## Supplemental Material

**Files in this Data Supplement:**

- Supplemental Material.docx
