# Supplementary material for: Thermodynamic stabilities of three-way junction nanomotifs in prohead RNA
Source: RNA. 2017 Apr;23(4):521–9. doi: 10.1261/rna.059220.116 (PMC5340915; doi:10.1261/rna.059220.116)
Supplement: Supplemental Material [file supp_059220.116_Supplemental_Material.docx]

***Supporting Information for thermodynamic stabilities of three-way junction nanomotifs in prohead RNA***

*Alyssa C. Hill^†^ and Susan J. Schroeder*^†‡^*

^†^Department of Microbiology and Plant Biology, ^‡^Department of Chemistry and Biochemistry, University of Oklahoma, Norman, Oklahoma 73019, United States

*Corresponding author e-mail address: susan.schroeder@ou.edu

**Figure S1.** Single-stranded optical melting data.





**Figure S2.** Pairwise optical melting data.

**

**

**Table S1.** Comparison of predicted and experimental free energies.

| Sequence | *∆G_37_* (kcal mol^-1^) | | | | | | | | | |
| --- | --- | --- | --- | --- | --- | --- | --- | --- | --- | --- |
|  | RNA Structure | | RNAfold | | mfold | | RNAsoft | | Experimental | |
|  | Construct | 3WJ | Construct | 3WJ | Construct | 3WJ | Construct | 3WJ | Construct | 3WJ |
| Phi29 | -29.2 | 1.9 | -28.5 | 2.6 | -30.1 | 1.0 | -29.0 | 2.1 | -26.5 | 4.6 |
| GA1 | -28.1 | 1.7 | -29.3 | 0.5 | -29.5 | 0.3 | n/a* | n/a* | -27.9 | 1.9 |
| SF5 | -27.3 | 3.4 | -27.4 | 3.3 | -28.3 | 2.4 | -26.8 | 3.9 | -35.0 | -4.3 |
| M2 | -31.7 | -1.7 | -31.4 | -1.4 | -33.0 | -3.0 | -32.3 | -2.3 | -39.9 | -9.9 |
| Phi29∆U29 | -29.1 | 2.0 | -29.1 | 2.0 | -29.5 | 1.6 | -28.4 | 2.7 | -25.8 | 5.3 |
| Phi29∆U29/∆U72 | -29.1 | 2.0 | -29.1 | 2.0 | -29.5 | 1.6 | -28.4 | 2.7 | -25.8 | 5.3 |
| Phi29∆U29/∆U72-73 | -29.1 | 2.0 | -29.1 | 2.0 | -29.3 | 1.8 | -28.2 | 2.9 | -34.4 | -3.3 |
| Phi29∆U29/∆U72-73-74 | -28.9 | 2.2 | -29.8 | 1.3 | -29.0 | 2.1 | -27.9 | 3.2 | -30.5 | 0.6 |
| Phi29∆U72 | -29.2 | 1.9 | -28.5 | 2.6 | -30.1 | 1.0 | -29.0 | 2.1 | -26.4 | 4.7 |
| Phi29∆U72-73 | -29.0 | 2.1 | -28.5 | 2.6 | -29.9 | 1.2 | -28.8 | 2.3 | -20.9 | 10.2 |
| Phi29∆U72-73-74 | -28.8 | 2.3 | -28.9 | 2.2 | -29.6 | 1.5 | -28.5 | 2.6 | -25.8 | 5.3 |

**Table S1.** Calculated secondary structure stabilities for pRNA 3WJs. For predictions in RNA Structure, RNAfold, and mfold, pRNA strands 3WJa and 3WJb as well as 3WJb and 3WJc were joined with a 5' – aaaa – 3' hairpin and then the construct was folded as a single strand. To correct for the added hairpins, two 5' – aaaa – 3' hairpin penalties and three initiation terms were subtracted from the secondary structure stabilities output by RNA Structure, RNAfold, and mfold. For stabilities predicted by RNAsoft, one 5' – aaaa – 3' hairpin penalty and two initiation terms were subtracted. *RNAsoft did not output a secondary structure for the GA1 pRNA 3WJ construct due to computational time limitations.

**Table S2.** Thermodynamic parameters for pRNA constructs with metal ions.

| Condition | *∆H* (kcal mol^-1^) | | | | *∆S* (cal mol^-1^ K^-1^) | | | | *∆G*_37_ (kcal mol^-1^) | | | |
| --- | --- | --- | --- | --- | --- | --- | --- | --- | --- | --- | --- | --- |
|  | Phi29 | GA1 | SF5 | M2 | Phi29 | GA1 | SF5 | M2 | Phi29 | GA1 | SF5 | M2 |
| 1 M Na^+^ | -231.6 | -358.2 | -308.1 | -354.2 | -663.5 | -1055.6 | -889.0 | -1015.9 | -25.8 | -30.8 | -32.3 | -39.1 |
| 100 mM Na^+^ | -226.7 | -278.8 | -189.7 | -167.4 | -655.0 | -840.6 | -560.9 | -478.3 | -23.5 | -18.1 | -15.7 | -19.0 |
| 100 mM Na^+^  10 mM Mg^2+^ | -223.2 | n/a* | -292.5 | -310.5 | -639.2 | n/a* | -851.7 | -880.7 | -25.0 | n/a* | -28.3 | -37.4 |
| 100 mM Na^+^  10 mM spermidine | -235.9 | -311.1 | -540.8 | -604.7 | -679.7 | -900.8 | -1594.9 | -1746.5 | -25.1 | -31.7 | -46.2 | -63.1 |

*****Optical melts of the GA1 3WJ in 100 mM Na^+^ and 10 mM Mg^2+^ did not meet the van’t Hoff plot goodness of linear fit cutoff criterion of ≥ 0.90.

**Figure S3.** Gel mobility of RNA single strands, pairwise combinations, and 3WJs in TMS buffer.


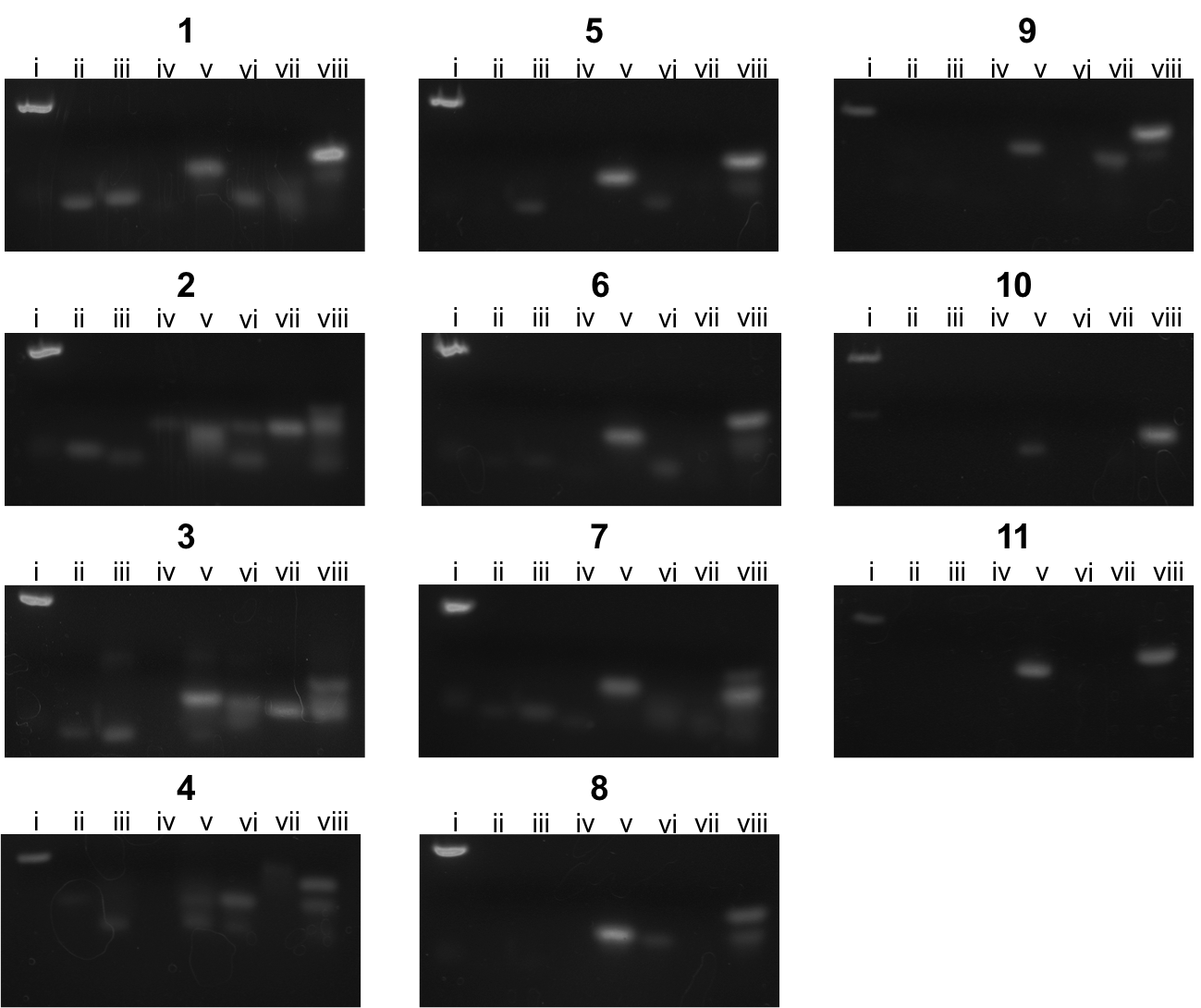


**Figure S3.** Gel mobility of 100 bp ladder (Lane i); individual strands 3WJa (Lane ii), 3WJb (Lane iii), and 3WJc (Lane iv); pairwise strands 3WJa + 3WJb (Lane v), 3WJb + 3WJc (Lane vi), and 3WJc + 3WJa (Lane vii); and strands 3WJa + 3WJb + 3WJc (Lane viii). 1: Phi29, 2: GA1, 3: SF5, 4: M2, 5: Phi29∆U29, 6: Phi29∆U29/∆U72, 7: Phi29∆U29/∆U72-73, 8: Phi29∆U29/∆U72-73-74, 9: Phi29∆U72, 10: Phi29∆U72-73, 11: Phi29∆U72-73-74. Assembly was performed in TMS buffer (50 mM Tris-HCl, pH 7.8, 100 mM NaCl, 10 mM MgCl_2_) for the purpose of comparison to previous work published on the assembly and stabilities of various biological RNAs (Shu et al. 2011).

**Figure S4.** Possible species formed by strands 3WJa, 3WJb, and 3WJc pairwise (A, B, and C) and all together (D, E, F, and G).


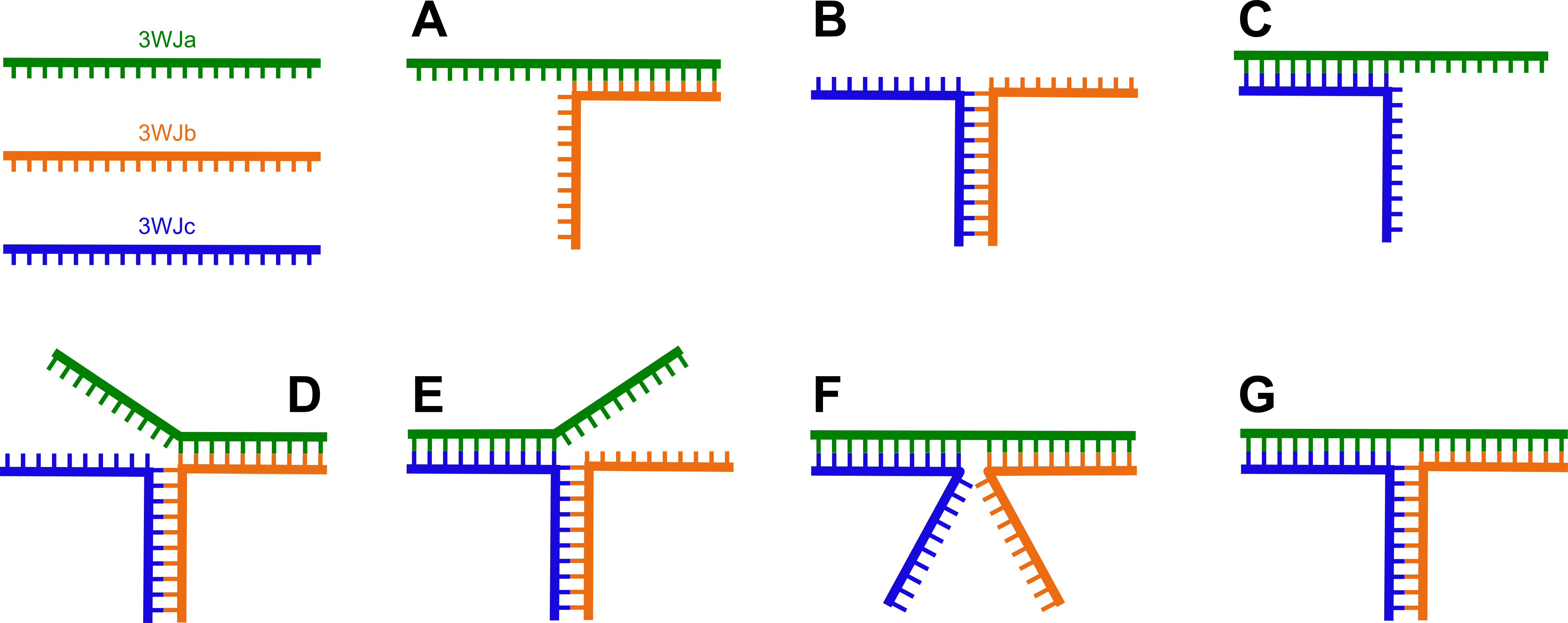


**Table S3.** Calculated ∆G_37_ and ∆H values from the Nearest Neighbor Database (Turner and Mathews 2010) for possible species formed by phi29, SF5, and M2 strands 3WJa, 3WJb, and 3WJc (see Figure S4) compared to experimental values.

| Species | | Helices formed | Phi29 | | SF5 | | M2 | |
| --- | --- | --- | --- | --- | --- | --- | --- | --- |
|  |  |  | ∆G_37_ (kcal/mol) | ∆H (kcal/mol) | ∆G_37_ (kcal/mol) | ∆H (kcal/mol) | ∆G_37_ (kcal/mol) | ∆H (kcal/mol) |
| A | Branch 1 | | -11.5 | -82.7 | -10.7 | -76.3 | -10.6 | -76.4 |
| B | Branch 2 | | -10.7 | -72.1 | -10.7 | -69.6 | -13.8 | -75.8 |
| C | Branch 3 | | -11.1 | -80.3 | -11.2 | -77.4 | -11.3 | -76.9 |
| D | Branches 1 and 2 | | -20.8 | -150.5 | -20.2 | -141.2 | -23.7 | -154.5 |
| E | Branches 2 and 3 | | -21.6 | -150.4 | -21.6 | -145.4 | -24.7 | -152.6 |
| F | Branches 3 and 1 | | -22.0 | -159.0 | -21.0 | -150.9 | -21.0 | -150.5 |
| G | Branches 1, 2, and 3 | | -27.5 | -207.6 | -26.8 | -206.7 | -30.4 | -221.0 |
| Experimental value | Branches 1, 2, and 3 | | -26.5 | -230.8 | -35.0 | -336.1 | -39.9 | -350.7 |

**Table S3.** Calculations for species A, B, and C include parameters for the formed helix, dangling ends, and one initiation penalty. Calculations for species D, E, and F include parameters for the formed helices, dangling ends, and two initiation penalties. The calculation for species G includes parameters for the formed helices, two initiation penalties, and a penalty for forming a 3-branch loop (Turner and Mathews 2010; Laing et al. 2011). Formation of the 3WJ is favored when all three strands are present As previously shown for RNA duplexes, even when the *T_m_* for a singlestrand forming a self-complementary duplex is higher, the non-self-complementary duplex will form as long as the enthalpy for the non-self-complementary duplex is more favorable, (Schroeder and Turner 2000).

**Equation S1.** Derivation of *Keq*.

1. $K_{eq}= \frac{[ABC]}{\left[ A \right]\left[ B \right][C]}$
2. $C_{T}=total strand concentration, \alpha=fraction in RNA triplex$
3. $K_{eq}= \frac{\frac{1}{3}C_{T}\alpha}{{[\frac{1}{3}C_{T}\left( 1- \alpha\right)]}^{3}}$
4. $K_{eq}= \frac{\frac{1}{3}C_{T}\alpha}{\frac{1}{27}{C_{T}}^{3}{(1- \alpha)}^{3}}$
5. $K_{eq}= \frac{9\alpha}{{C_{T}}^{3}\left( 1- \alpha\right)^{3}}$
6. $At T_{m}, \alpha=0.5$
7. $K_{eq}= \frac{9(0.5)}{{C_{T}}^{2}{(0.5)}^{3}}$
8. $K_{eq}= \frac{36}{{C_{T}}^{2}}$

***References***

Laing C, Wen D, Wang JTL, Schlick T. 2011. Predicting coaxial helical stacking in RNA junctions. *Nucleic Acids Research*.

Schroeder SJ, Turner DH. 2000. Factors affecting the thermodynamic stability of small asymmetric internal loops in RNA. *Biochemistry* **39**: 9257-9274.

Shu D, Shu Y, Haque F, Abdelmawla S, Guo P. 2011. Thermodynamically stable RNA three-way junction for constructing multifunctional nanoparticles for delivery of therapeutics. *Nature Nanotechnology* **6**: 658-667.

Turner DH, Mathews DH. 2010. NNDB: the nearest neighbor parameter database for predicting stability of nucleic acid secondary structure. *Nucleic Acids Research* **38**: D280-D282.
